# Agenesis of the Dorsal Pancreas: A Rare Cause of Diabetes and Recurrent Upper Abdominal Pain

**DOI:** 10.7759/cureus.34689

**Published:** 2023-02-06

**Authors:** Ankit Lalchandani, Ajeet Maurya, Syed Fazal Mehdi Rizvi, Amit Yadav

**Affiliations:** 1 General Surgery, All India Institute of Medical Sciences Bhopal, Bhopal, IND

**Keywords:** recurrent abdominal pain, type 1 diabetes mellitus, polysplenia, pancreatic anomaly, agenesis of dorsal pancreas, heterotaxy syndrome, congenital anomaly, pancreas agenesis

## Abstract

Agenesis of the dorsal pancreas is a rare congenital disorder with only a handful of cases described in the literature. It presents a diagnostic dilemma. Cross-sectional imaging is the cornerstone for diagnosis. It could have a syndromic association with polysplenia and cardiac anomalies. Pancreas divisum and chronic pancreatitis may present with similar symptoms and must be ruled out. We present a case of a 55-year-old male with recurrent non-specific abdominal pain and diabetes mellitus. He was managed with insulin and painkillers for symptomatic relief. We also reviewed approximately 68 cases described in the literature to date.

## Introduction

Agenesis of the dorsal pancreas is a rare congenital disorder that usually presents in early adulthood. The symptoms are non-specific and create a diagnostic dilemma. Less than a hundred have been described in the literature to date [1]. It may be at times associated with other anomalies, such as polysplenia and cardiac anomalies, and has been thought to be part of a syndrome [2]. We present a case of agenesis of the dorsal pancreas with a review of the literature.

## Case presentation

A 55-year-old male patient presented to us with a two-month history of pain over the epigastrium and right hypochondrium. It was dull, intermittent, non-radiating, and had no identifiable aggravating or relieving factors. There was no history of vomiting, jaundice, or fever. There were no symptoms suggestive of steatorrhea. However, the patient had chronic constipation. He also had a history of recurrent urinary tract infections associated with burning micturition. He sought treatment at a private setup and was treated with oral medications. He could not provide any previous urine routine microscopy and culture reports. He was a known diabetic on oral hypoglycaemic agents for the past six years, which were started at a private clinic. He was taking glimepiride 1 mg once a day and metformin 500 mg twice a day. On presentation, the patient's blood pressure was 110/70 mmHg, pulse rate was 94 bpm, respiratory rate was 16 pm, saturation level was 97% on room air, and he was afebrile. His blood sugar level, as measured by a glucometer, was 330 mg/dl. On general examination, the patient was thin-built and had mild pallor. The abdomen examination was essentially normal. The rectal and proctoscopic examination revealed grade two internal hemorrhoids. Routine blood investigations, including amylase and lipase, were within normal limits except for glycosylated hemoglobin (HbA1c), which was 9.6% indicating poorly controlled diabetes. 

**Figure 1:**
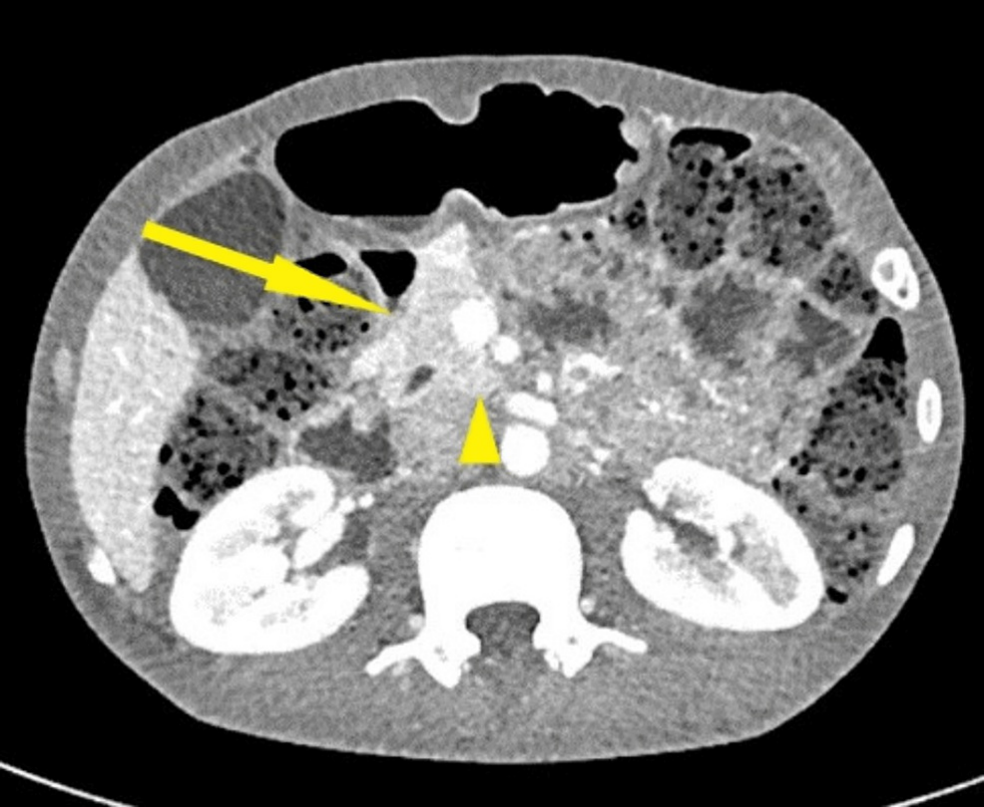
CECT showing the head (solid arrow) and uncinate process (arrowhead) of the pancreas CECT: contrast-enhanced computed tomography

**Figure 2:**
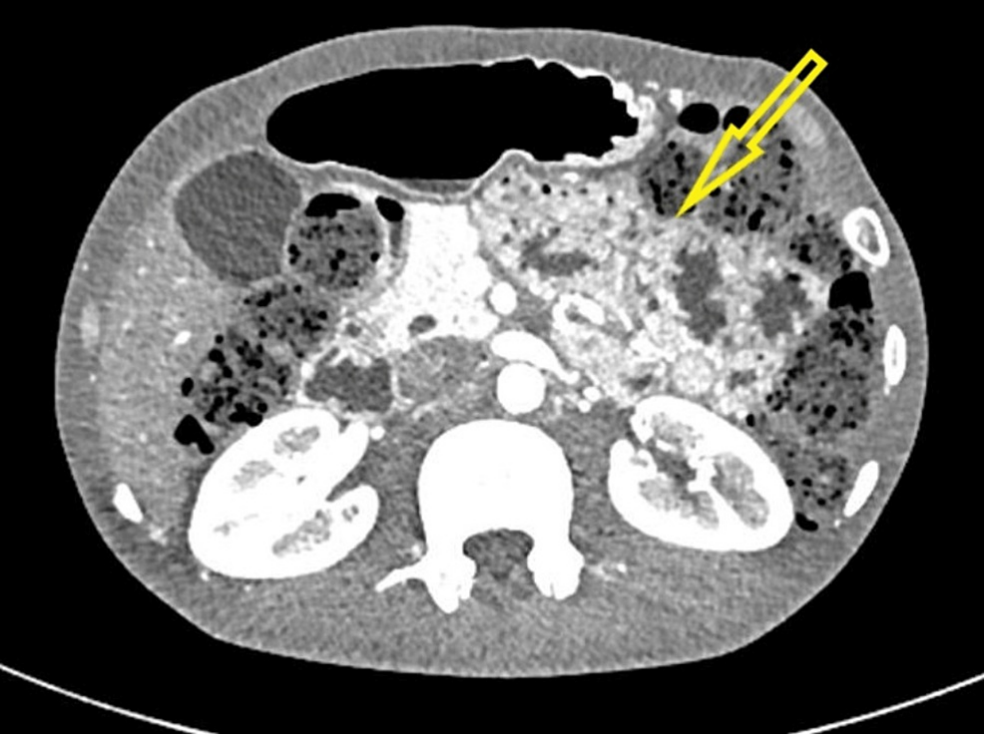
CECT of the abdomen showing small bowel loops occupying the region of the pancreatic body and tail (hollow arrow) CECT: contrast-enhanced computed tomography

**Figure 3:**
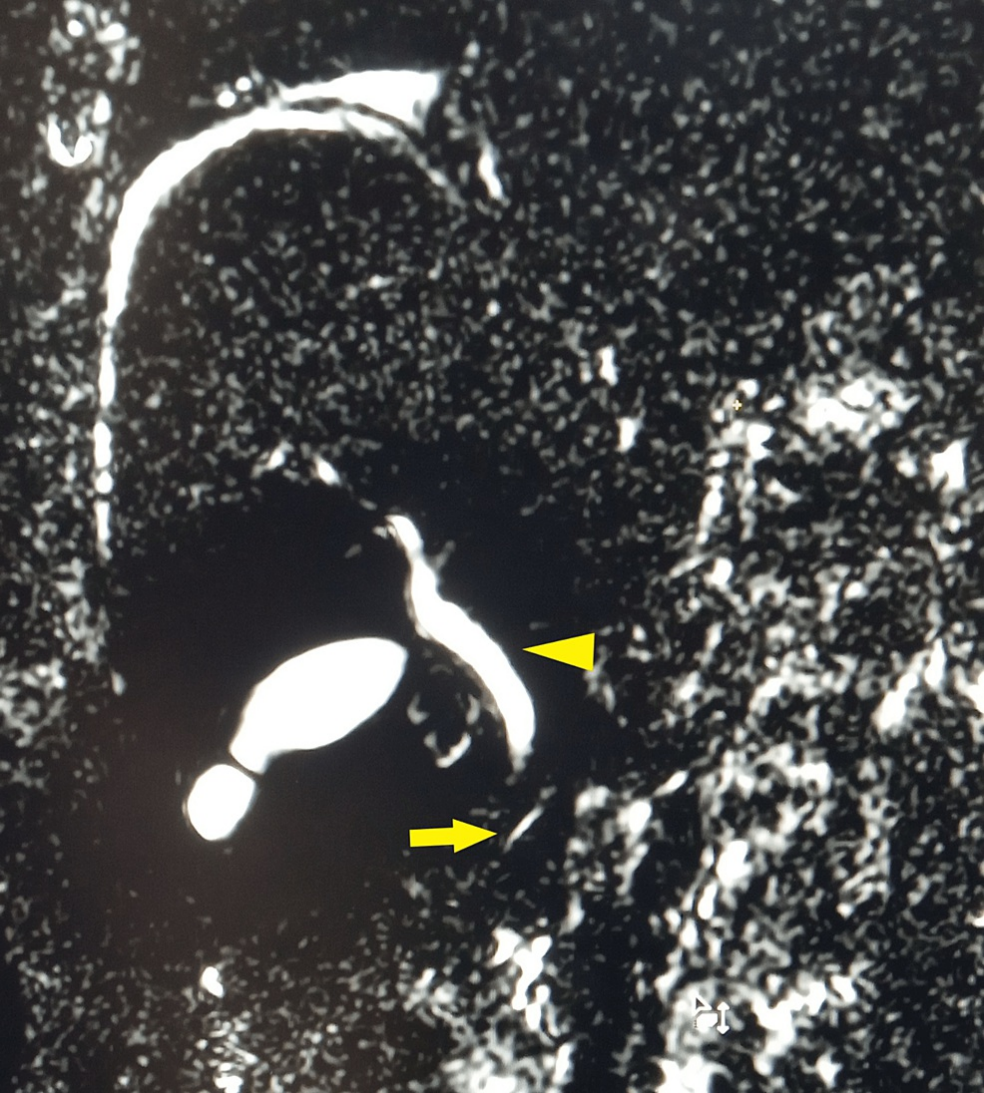
MRCP showing the presence of ventral pancreatic duct (arrow) and common bile duct (arrowhead) but no dorsal pancreatic duct MRCP: magnetic resonance cholangiopancreatography

The patient was started on intermediate-acting insulin in the ward. The blood sugar levels were gradually brought under control with a daily requirement of 20 U of intermediate-acting insulin. Contrast-enhanced computed tomography of the abdomen was done, which demonstrated non-visualization of the neck, body, and tail of the pancreas suggesting dorsal pancreatic agenesis (Figure 1). The visualized part of the pancreas appeared normal. The region of the body and tail was occupied by small bowel loops (Figure 2). Subsequently, a magnetic resonance cholangiopancreatography was done, which suggested the absence of a dorsal pancreatic duct. A small ventral pancreatic duct was visualized draining into the ampulla of Vater (Figure 3). The patient was started on laxatives to relieve constipation. Over a five-day period of admission, the patient developed adequate blood sugar control and pain relief and was discharged on intermediate-acting insulin and laxatives. The patient did not show up for a follow-up subsequently.

## Discussion

The pancreas arises from the endoderm via the dorsal and ventral buds, which form as outpouchings from the foregut. The signaling pathways include the hedgehog, PDX1, and NOTCH genes. The dorsal bud develops more rapidly and forms the neck, body, and tail of the pancreas drained by the duct of Santorini. The ventral bud rotates clockwise to fuse with the dorsal segment, ultimately forming the head and uncinate process drained by the duct of Wirsung [3].

Agenesis of the dorsal pancreas is an extremely rare disorder. We did the literature search on PubMed Central, Europe PMC, Scopus, and Google Scholar using the keywords: 'genesis of dorsal pancreas' and 'dorsal pancreatic agenesis'. A total of 57 publications were identified describing a total of 68 cases from 1913 to 2021 (Table 1). Among those described cases, there were 38 females and 30 males. The age range was one month to 79 years. Thirty-one (45%) patients either had a history of diabetes or presented with symptoms related to it. Thirty-one (45%) patients had recurrent upper abdominal pain as the presenting symptom. Seven (10%) had diarrhea. In 14 (20%) patients, the diagnosis was made incidentally on cross-sectional imaging or during autopsy studies. About 30 (44%) patients had associated anomalies, including polysplenia and cardiac and renal anomalies in decreasing order of frequency. These are thought to be a part of heterotaxy syndrome. Contrast-enhanced computed tomography was the mainstay of diagnosis and showed a normal pancreatic head and uncinate process and absence of pancreatic body and tail. The empty space was occupied by small bowel loops or the body of the stomach. Magnetic resonance cholangiopancreatography and endoscopic retrograde cholangiopancreatography showed an absent dorsal pancreatic duct. Patients were managed symptomatically with insulin and pancreatic enzyme supplementation wherever indicated.

**Table 1:** Review of case reports

Author	Age/sex	Presentation	Associated anomalies
Gohn [4]	14/M	Epigastric pain	Renal duplication, bilobar right lung, mental retardation
Priesel [5]	56/F	Asymptomatic	
Duschl [6]	21/M	Diabetes mellitus	
Kriss [7]	1 mo/M	Asymptomatic	
Lechner et al. [8]	26/M	Diabetes mellitus	
Gurson [9]	1 mo/F	Asymptomatic	Ventricular septal defect
Sano et al. [10]	40/M	Diabetes mellitus	Spina bifida
Shimaguchi et al. [11]	54/M	Diabetes mellitus	
Gilinsky et al. [12]	40/F	Epigastric pain	
	32/M	Diabetes mellitus	
Shah et al. [13]	32/F	Epigastric pain, diabetes mellitus	
Lehman et al. [14]	47/F	Epigastric pain	
Bretangne et al. [15]	25/M	Epigastric pain, diabetes mellitus	
Nishimori et al. [16]	32/F	Epigastric pain	Vaginal atresia, atrophic gastritis
Wang et al. [17]	54/M	Diabetes mellitus	
Herman et al. [18]	8/M	Meningitis	Polysplenia, congenital heart disease, absent left kidney
Soler et al. [19]	48/F	Diabetes mellitus	Polysplenia, uterine leiomyomas, prominent azygous vein
	68/F	Lower quadrant pain	Polysplenia, enlarged azygous vein
Wildling et al. [20]	12/M	Asymptomatic	
	16/M	Asymptomatic	
	39/F	Epigastric pain, diabetes mellitus, diarrhea	
Gold [21]	70/F	Epigastric pain, diabetes mellitus	
Klein et al. [22]	46/F	Diabetes mellitus, diarrhea	
Deignan et al. [23]	64/M	Epigastric pain	
Oldenberg et al. [24]	39/F	Epigastric pain, diabetes mellitus	
Macari et al. [25]	46/F	Epigastric pain	
Teruzzi et al. [26]	62/M	Epigastric pain	
Fukuoka et al. [27]	47/F	Obstructive jaundice	
Nakamura et al. [28]	28/F	Epigastric pain	Solid pseudopapillary tumor
Nonent et al. [29]	73/F	Lower abdominal pain	Carcinoma ovary
Guclu et al. [30]	33/F	Epigastric pain, diabetes mellitus	
Ulusan et al. [31]	49/F	Epigastric pain	Solid pseudopapillary tumor
Otani et al. [32]	59/F	Epigastric pain	Choledochal cyst
Ashraf et al. [33]	1 mo/M	Diabetes mellitus	Cardiac septal defect, gall bladder agenesis, malrotation
Sempere et al. [34]	23/F	Epigastric pain	Pancreatic pseudocyst, dilated main pancreatic duct
Joo et al. [35]	25/F	Epigastric pain, diabetes mellitus	
Kapa et al. [36]	25/M	Asymptomatic	Coarctation of aorta (heterotaxy syndrome)
Haldorsen et al. [37]	6/F	Diarrhea	Renal abnormality
	35/F	Diabetes mellitus, diarrhea	Renal abnormality
	62/F	Diabetes mellitus, diarrhea	Renal abnormality
	15/M	Diarrhea	
	38/M	Diabetes mellitus, diarrhea	
Balakrishna et al. [38]	28/F	Epigastric pain	
Pasaoglu et al. [39]	62/F	Asymptomatic	
Mohapatra et al. [40]	30/M	Epigastric pain	
	30/M	Lower abdominal pain	
Gagniere et al. [41]	36/F	Asymptomatic	Mucinous cystic neoplasm pancreatic head
Thakur et al. [42]	42/M	Epigastric pain	Cholelithiasis
Robert et al. [43]	34/M	Asymptomatic	
Jung et al. [44]	13/F	Diabetes mellitus	Double outlet right ventricle (heterotaxy syndrome)
Liang et al. [45]	23/F	Diabetes mellitus	
Jain et al. [1]	35/F	Epigastric pain, diabetes mellitus	
Rodrigues et al. [46]	48/F	Asymptomatic	Pancreatic neuroendocrine tumor
Kabnurkar et al. [47]	49/M	Asymptomatic	Carcinoma tongue
Sonkar et al. [48]	25/M	Epigastric pain, diabetes mellitus	Skeletal deformity, scoliosis
Erotokrito et al. [49]	71/M	Epigastric pain, diabetes mellitus	pancreatic neuroendocrine tumor
Riguetto et al. [50]	40/F	Diabetes mellitus	Polysplenia (heterotaxy syndrome)
Zhongh et al. [51]	67/F	Asymptomatic	
Yang et al. [52]	30/M	Epigastric pain, diabetes mellitus	
Bhandari et al. [53]	17/F	Asymptomatic	Unilateral renal agenesis, unicornuate uterus, ectopic right ovary
Ustabasiog et al. [54]	17/F	Epigastric pain, diabetes mellitus	Pancreatic cyst
Mei et al. [55]	61/F	Epigastric pain, diabetes mellitus	
	65/F	Epigastric pain, diabetes mellitus	
Dinkhauser et al. [56]	65/F	Epigastric pain, diabetes mellitus	
Valiyeva et al. [57]	73/M	Epigastric pain, diabetes mellitus	Choledocholithiasis
	79/M	Asymptomatic	Carcinoma bladder
Chapa et al. [58]	23/M	Epigastric pain	Hirschprung, choledochal cyst
Xia et al. [59]	51/M	Jaundice	

## Conclusions

Agenesis of the dorsal pancreas presents a diagnostic challenge as most of these patients present with non-specific abdominal symptoms. One of the major differential diagnoses to be ruled out is pancreas divisum. Cross-sectional imaging and endoscopic retrograde cholangiopancreatography will show the separation of the ventral and dorsal pancreatic ductal system. Atrophy of the body and tail of the pancreas occurs in chronic pancreatitis and may mimic agenesis. In such a scenario, there may be a history of previous abdominal pain radiating to the back associated with raised amylase and lipase levels. Agenesis of the dorsal pancreas has also been shown to have a syndromic association with polysplenia and the annular pancreas. Since presenting symptoms are non-specific, cross-sectional imaging and magnetic resonance cholangiopancreatography are the cornerstones for diagnosis. There is no specific treatment for this rare disorder. Patients need to be offered symptomatic management and diabetes control with insulin preparations. Steatorrhea due to exocrine insufficiency may be present, which can be managed by enzyme supplementation.
